# Deubiquitinases in cancer: biology, mechanism, and therapeutic targeting

**DOI:** 10.1016/j.bbrep.2026.102877

**Published:** 2026-11-07

**Authors:** Mohd Altaf Najar, Prashant Kumar Modi, Raja Rajeshwari

**Affiliations:** Center for Systems Biology and Molecular Medicine, Yenepoya Research Centre, Yenepoya (Deemed to be University), Mangalore, 575018, India

**Keywords:** Deubiquitinases, Ubiquitin signaling, Post-translational modification, Cell cycle regulation, DNA damage response, Proteostasis

## Abstract

The ubiquitin-proteasome system (UPS) plays a central role in cellular protein homeostasis by regulating the degradation, trafficking, and activity of target proteins. Deubiquitinases (DUBs) are a crucial component of this regulatory network, catalyzing the removal of ubiquitin moieties from modified substrates to modulate signaling pathways that govern cell cycle control, apoptosis, DNA repair, immune responses, and stress adaptation. Aberrant DUB expression or activity is increasingly recognized as a driver of oncogenesis, tumor progression, drug resistance, and metastasis. As a result, DUBs have emerged as novel therapeutic targets in cancer, complementing existing proteasome inhibitors. This review provides an overview of DUB classification, their mechanistic roles in cancer biology, current progress in small-molecule DUB inhibitors and DUB-directed PROTACs, and discusses translational challenges and future opportunities for therapeutic development.

## Highlights

• Deubiquitinases regulate cancer signaling, proteostasis, and genome stability.
• Dysregulated DUB activity promotes tumor progression and therapy resistance.
• DUBs modulate key oncogenic pathways including p53, NF-κB, and PI3K-AKT.
• Emerging DUB inhibitors and DUBTACs show therapeutic potential in cancer.
• Precision targeting of ubiquitin signaling represents a promising anticancer strategy.

## 1 Introduction

Protein homeostasis is fundamental to maintaining cellular integrity and ensuring appropriate responses to developmental, physiological, and environmental cues [1], [2]. Among the regulatory systems that govern proteome dynamics, the ubiquitin-proteasome system (UPS) has emerged as one of the most sophisticated mechanisms controlling protein stability, activity, localization, and intermolecular interactions. Through the sequential action of ubiquitin-activating (E1), ubiquitin-conjugating (E2), and ubiquitin ligase (E3) enzymes, ubiquitin is covalently attached to substrate proteins, thereby regulating diverse cellular processes beyond proteasomal degradation, including DNA damage repair, cell cycle progression, transcriptional regulation, signal transduction, autophagy, chromatin remodeling, immune responses, and cellular metabolism. [3], [4]. Ubiquitination determines protein fate based on chain topologyK48-linked polyubiquitin marks proteins for proteasomal degradation, while other chain linkages (e.g., K63, K11, K6) regulate signaling, DNA repair, and autophagy [5], [6], [7]. Dysregulation of ubiquitin signaling disrupts these tightly coordinated processes and contributes to the initiation and progression of numerous human diseases, particularly cancer, where aberrant protein turnover promotes malignant transformation, therapeutic resistance, and tumor evolution.

Deubiquitinases (DUBs) counteract ubiquitination by cleaving ubiquitin chains from substrate proteins [8], [9], [10] rescuing them from degradation, modulating their localization and stability, and recycling ubiquitin monomers. More than 100 human deubiquitinases (DUBs) have been identified and are currently classified into seven evolutionarily and structurally distinct families based on their catalytic mechanisms and conserved domain architectures: ubiquitin-specific proteases (USPs), ubiquitin C-terminal hydrolases (UCHs), ovarian tumor proteases (OTUs), Machado-Joseph disease proteases (MJDs/Josephins), JAB1/MPN/MOV34 metalloproteases (JAMMs), motif interacting with ubiquitin-containing novel DUB family (MINDYs), and zinc finger-containing ubiquitin peptidase 1 (ZUFSP, also known as ZUP1). With the exception of the JAMM family, which comprises zinc-dependent metalloproteases, all human DUB families function as cysteine proteases. Together, these enzymes regulate ubiquitin chain editing, substrate stabilization, and ubiquitin recycling, thereby maintaining cellular proteostasis and signaling fidelity [11]. Dysregulated DUB activity contributes to malignant transformation by stabilizing oncogenic factors or suppressing tumor suppressors [12], [13], [14], [15], [16]. A schematic overview of the ubiquitin-proteasome system, highlighting E1, E2, E3-mediated ubiquitination, proteasomal degradation, and the opposing role of deubiquitinases, is shown in Fig. 1.

**Fig. 1:**
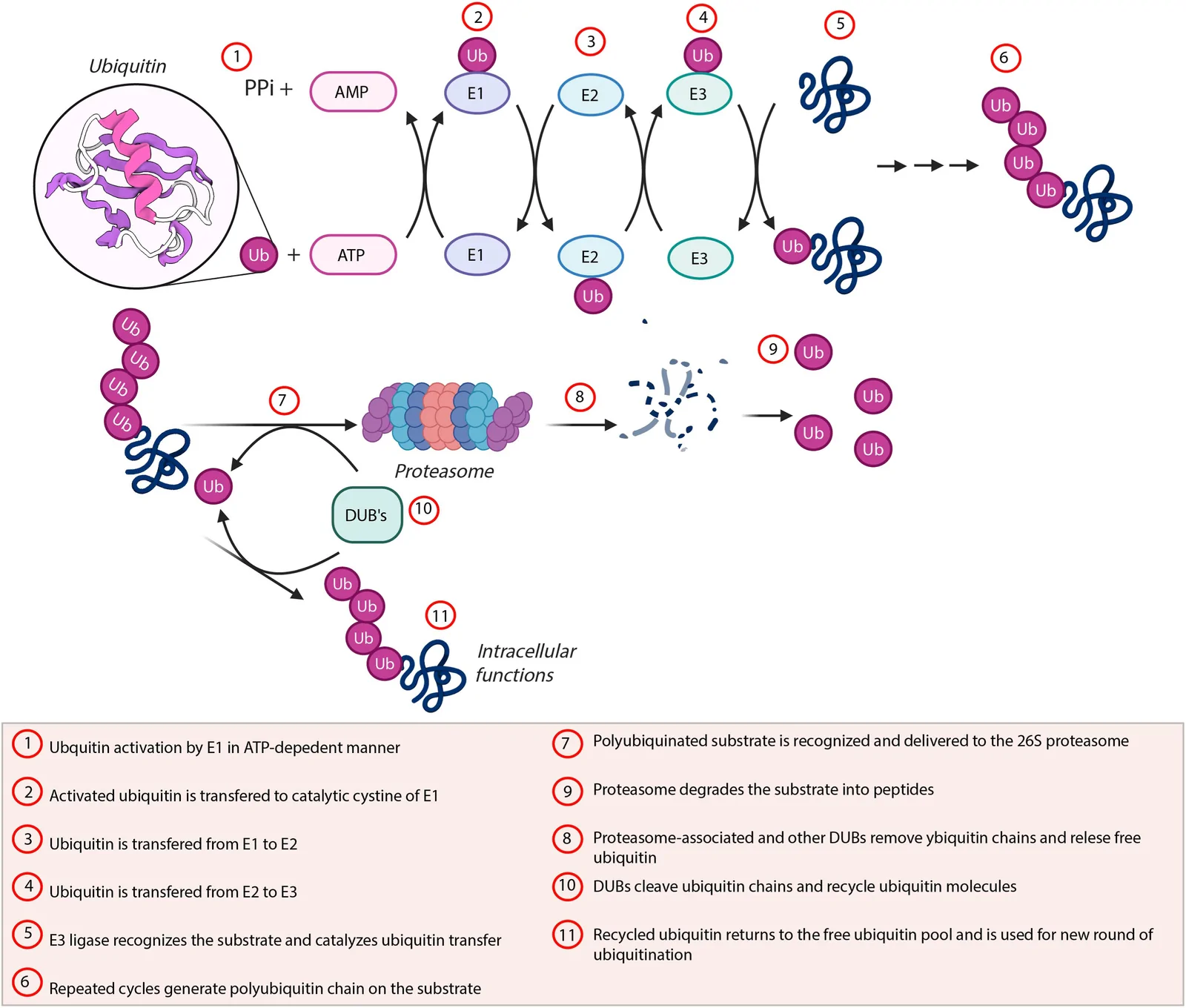
**Overview of the ubiquitin-proteasome system (UPS) and DUB-mediated ubiquitin recycling:** The numbered arrows indicate the sequential steps: (1) ATP-dependent ubiquitin activation by E1; (2) transfer of ubiquitin to the E1 catalytic cysteine; (3) transfer from E1 to E2; (4) transfer from E2 to an E3-associated substrate; (5) E3-mediated ubiquitin conjugation to the substrate; (6) iterative ubiquitination to form a polyubiquitin chain; (7) recognition of the polyubiquitinated substrate by the 26S proteasome; (8) removal of ubiquitin chains by proteasome-associated DUBs; (9) proteolytic degradation of the substrate; (10) further ubiquitin-chain processing and recycling by DUBs; and (11) return of free ubiquitin to the cellular ubiquitin pool.

Growing recognition that DUBs orchestrate multiple oncogenic signaling networks including p53-MDM2 regulation, NF-κB signaling, DNA damage repair, apoptosis resistance, and immune escape has stimulated intense research into DUB inhibitors and protein-degradation-based strategies such as PROTACs [17], [18], [19], [20]. Unlike conventional proteasome inhibitors (e.g., bortezomib), which indiscriminately impair global protein degradation, targeting individual DUBs offers the opportunity to modulate specific ubiquitin-dependent signaling pathways, thereby improving therapeutic precision while potentially minimizing systemic toxicity [9], [21], [22]. These advances reflect a broader shift from global inhibition of the ubiquitin-proteasome system toward precise manipulation of ubiquitin signaling networks, positioning DUBs as promising targets for next-generation precision oncology.

Despite remarkable advances in understanding DUB biology, several important challenges remain. The context-dependent functions of individual DUBs, extensive substrate overlap, limited availability of highly selective inhibitors, and the complexity of ubiquitin signaling continue to hinder clinical translation. At the same time, rapid progress in structural biology, chemical biology, and targeted protein degradation has significantly expanded our understanding of DUB function and therapeutic potential. Therefore, an updated synthesis of recent discoveries is both timely and necessary. In this review, we critically discuss the structural classification, biological functions, and roles of DUBs in cancer, evaluate current therapeutic strategies, and highlight emerging opportunities and future challenges for translating DUB biology into precision oncology.

## 2 Classification and biological functions of DUBs

Deubiquitinases (DUBs) constitute a large and heterogeneous class of proteases responsible for removing ubiquitin moieties from protein substrates or editing polyubiquitin chains to regulate protein turnover, localization, and signaling. More than 100 human DUBs have been identified and are classified into seven evolutionarily conserved families based on their catalytic mechanisms and structural organization [11], [23]. These include the ubiquitin-specific proteases (USPs), ubiquitin C-terminal hydrolases (UCHs), ovarian tumor proteases (OTUs), Machado–Joseph disease proteases (MJDs/Josephins), JAB1/MPN/MOV34 metalloproteases (JAMMs), motif interacting with ubiquitin-containing novel DUB family (MINDYs), and zinc finger-containing ubiquitin peptidase 1 (ZUFSP/ZUP1). USPs constitute the largest DUB family, comprising nearly 60 members that regulate diverse processes including cell-cycle progression, DNA repair, transcription, and proteostasis [24], [25]. UCH family members primarily process ubiquitin precursors and recycle monoubiquitin, thereby maintaining ubiquitin homeostasis [26]. OTU DUBs exhibit remarkable ubiquitin-chain linkage specificity, enabling precise regulation of immune and inflammatory signaling pathways. MJD/Josephin DUBs participate in protein quality control and are implicated in neurodegenerative diseases such as spinocerebellar ataxia [27]. Unlike other DUB families, JAMM proteins are zinc-dependent metalloproteases that function within multiprotein complexes involved in proteostasis, membrane trafficking, and autophagy. MINDY and ZUFSP family members further expand the functional diversity of human DUBs through their distinct catalytic architectures and selective recognition of specific ubiquitin chain architectures [28]. Together, the seven DUB families provide the structural and catalytic framework that underpins the remarkable functional diversity of ubiquitin signaling [29]. DUB families are classified primarily according to their catalytic mechanisms and characteristic structural/domain features, whereas their biological functions are diverse and context-dependent; therefore, the functional annotations in Table 1 are representative rather than defining features of individual DUB families. An overview of the seven human DUB families, their catalytic mechanisms, conserved domain architectures, representative members, and representative biological functions is provided in Fig. 2 and Table 1.

**Table 1:** Classification of DUBs.

DUB Family	Catalytic Mechanism	Catalytic Domain	Representative Members	Chain Preference	Representative Biological Functions
USP (Ubiquitin-Specific Proteases)	Cysteine protease	USP domain	USP1, USP2, USP4, USP5, USP7, USP9X, USP10, USP14, USP15, USP22, USP28, USP36	Broad specificity	Largest DUB family; regulates cell-cycle progression, DNA damage repair, transcription, apoptosis, chromatin remodeling, immune signaling, and protein quality control.
UCH (Ubiquitin C-terminal Hydrolases)	Cysteine protease	UCH domain	UCHL1, UCHL3, UCHL5 (UCH37), BAP1	Mono-Ub/short chains	Processes ubiquitin precursors and ubiquitin adducts, maintains the free ubiquitin pool, regulates neuronal function, chromatin organization, and tumor suppression.
OTU (Ovarian Tumor DUBs)	Cysteine protease	OTU domain	OTUB1, OTUD1, OTUD3, OTUD5, OTUD6B, A20 (TNFAIP3), Cezanne (OTUD7B), OTULIN	K48, K63, M1 (family member-dependent)	Exhibits remarkable ubiquitin-chain linkage specificity (K48, K63, M1); regulates innate immunity, NF-κB signaling, DNA damage response, inflammation, and antiviral signaling.
MJD (Machado-Joseph Disease/Josephin Proteases)	Cysteine protease	Josephin domain	ATXN3, ATXN3L, JOSD1, JOSD2	Mixed linkage specificity	Regulates protein quality control, ubiquitin-chain editing, stress responses, and protein aggregate clearance; implicated in neurodegeneration and cancer.
JAMM (JAB1/MPN/MOV34 Metalloproteases)	Zinc-dependent metalloprotease	MPN+/JAMM domain	PSMD14 (RPN11), BRCC3 (BRCC36), STAMBP, STAMBPL1, CSN5	Broad; metalloprotease	Only metalloprotease DUB family; functions within multiprotein complexes to regulate proteasomal degradation, DNA repair, endosomal trafficking, autophagy, and receptor signaling.
MINDY (Motif Interacting with Ubiquitin-Containing Novel DUB Family)	Cysteine protease	MINDY domain	MINDY1, MINDY2, MINDY3, MINDY4,	Linkage-selective ubiquitin-chain editing, particularly K48-linked chains	
ZUFSP/ZUP1 (Zinc Finger-containing Ubiquitin Peptidase 1)	Cysteine protease	ZUFSP domain	ZUFSP/ZUP1	Preferential cleavage of K63-linked ubiquitin chains	regulation of DNA damage responses and ubiquitin signaling.

**Fig. 2:**
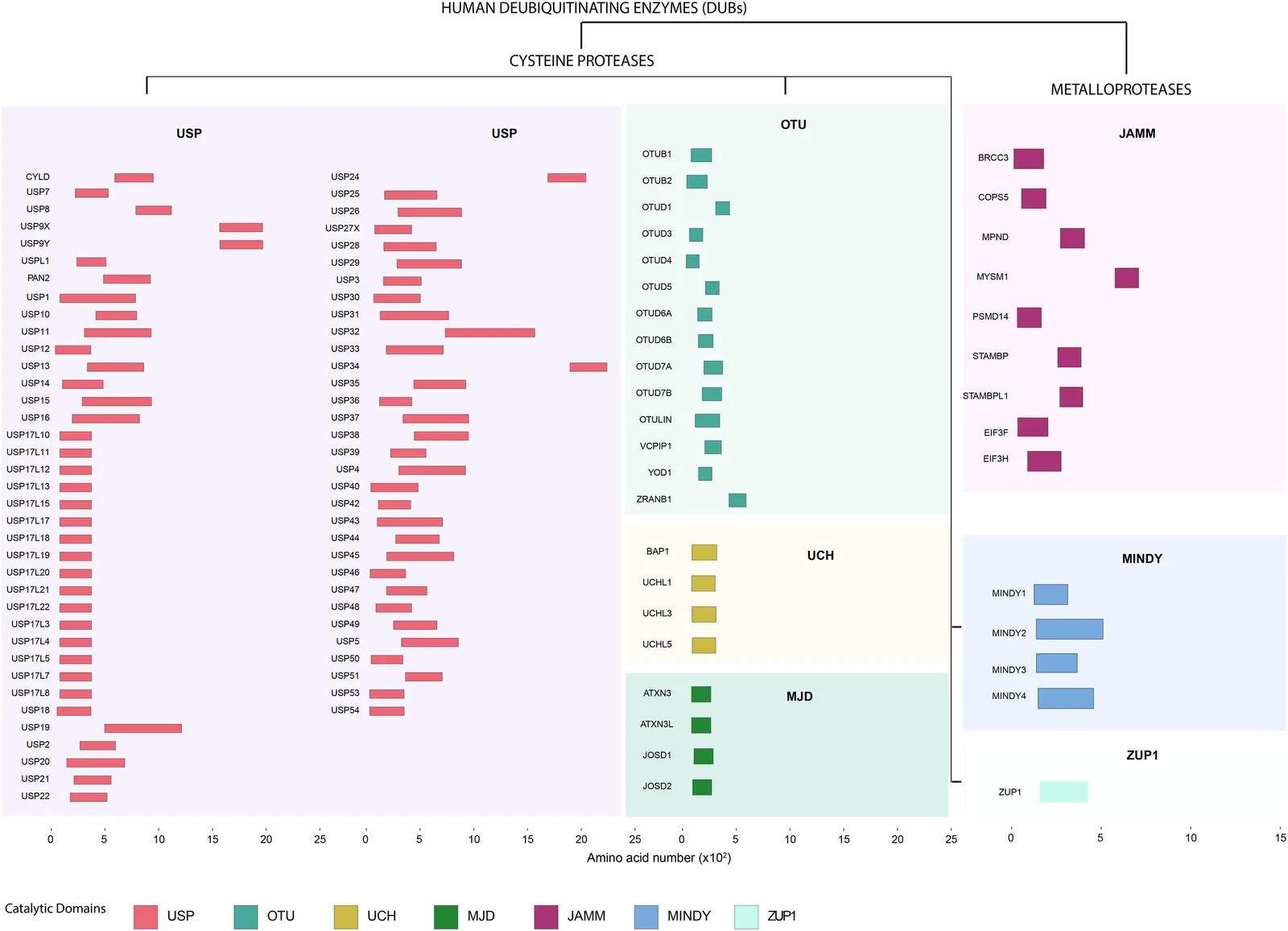
Domain-based classification of human deubiquitinating enzymes (DUBs): Human DUBs are grouped into seven major families USP, UCH, OTU, MJD, JAMM, MINDY, and ZUFSP (ZUP1) based on catalytic mechanism and domain architecture. Representative catalytic domains and family members are shown.

Beyond their structural diversity, DUBs are essential regulators of cellular homeostasis, fine-tuning ubiquitin-dependent processes including cell-cycle progression, DNA damage repair, transcription, apoptosis, chromatin remodeling, metabolism, and immune signaling. By selectively stabilizing oncogenes or tumor suppressors, DUBs exert context-dependent effects that can either promote or suppress tumorigenesis [30], [31]. Dysregulation of DUB expression or activity contributes to multiple hallmarks of cancer including replicative immortality, resistance to cell death, metastasis, angiogenesis, inflammation, and genomic instability [32]. Moreover, DUBs regulate innate and adaptive immune responses by modulating key signaling molecules such as TRAF6, NEMO, and RIG-I, thereby influencing inflammation, antiviral defense, and tumor immune evasion [33], [34]. Given their central role in both normal physiology and disease, DUBs have emerged as attractive therapeutic targets, with several inhibitors currently under preclinical and early clinical investigation [15], [35]. DUB activity is itself subject to multiple layers of regulation that ensure precise spatial and temporal control of ubiquitin signaling. Post-translational modifications (PTMs), including phosphorylation, ubiquitination, SUMOylation, acetylation, and redox-dependent oxidation, can modulate DUB catalytic activity, substrate specificity, protein stability, and intermolecular interactions. For example, phosphorylation regulates the activity and substrate recognition of several USP family members, whereas reversible oxidation of catalytic cysteine residues provides a mechanism for rapidly suppressing DUB activity during oxidative stress. In addition, the subcellular localization of DUBs critically determines substrate accessibility and biological function. Nuclear DUBs such as USP7 and BAP1 primarily regulate chromatin organization, DNA damage responses, and transcription, whereas proteasome-associated DUBs (USP14 and UCHL5) and organelle-specific DUBs such as USP30 control proteostasis and mitochondrial quality control, respectively. Many DUBs also require assembly into multiprotein complexes for full catalytic activity or substrate specificity. Representative examples include the USP1-UAF1 complex in DNA repair, the BAP1-ASXL (PR-DUB) complex in epigenetic regulation, and the BRCC36-containing BRCA1-A complex in DNA damage signaling. Dysregulation of these regulatory mechanisms can profoundly alter DUB activity, contributing to oncogenesis, therapeutic resistance, and immune evasion. Importantly, these regulatory networks also provide opportunities for developing highly selective therapeutic strategies that target DUB activation, localization, or protein-protein interactions rather than their conserved catalytic sites alone.

## 3 Mechanistic roles of DUBs

Deubiquitinases (DUBs) play essential mechanistic roles in preserving the integrity and balance of ubiquitin signaling by reversing ubiquitination events and thereby controlling the fate, stability, and function of cellular proteins. Mechanistically, DUBs remove ubiquitin from substrates through several modes of action: (i) cleaving mono-ubiquitin from proteins, (ii) trimming polyubiquitin chains from their distal ends, or (iii) editing chain architecture by selectively processing specific lysine-linked ubiquitin chains such as K48, K63, K11, or linear M1 linkages [36], [37]. DUBs also regulate substrate processing at the 26S proteasome, where the JAMM-family metalloprotease RPN11 removes ubiquitin chains to facilitate substrate degradation, whereas USP14 and UCHL5 can delay this process by trimming ubiquitin chains and rescuing selected proteins from degradation [38], [39], [40]. Beyond proteasomal degradation, DUBs also edit non-degradative ubiquitin signals, including K63-, K11-, and M1-linked chains, thereby regulating signaling activity, subcellular localization, protein–protein interactions, and scaffold assembly. Thus, DUB activity can influence both protein stability and ubiquitin-dependent signaling depending on the linkage type, substrate, and cellular context [41], [42]. DUBs also regulate diverse cellular processes including DNA damage repair, chromatin remodeling, transcription, cell-cycle progression, mitochondrial quality control, apoptosis, and immune signaling through substrate-specific deubiquitination [43], [44], [45]. Within immune signaling, DUBs such as A20, CYLD, and OTULIN maintain inflammatory balance by editing K63- or M1-linked chains on signaling adaptors including TRAF6, RIPK1, and NEMO, thereby constraining NF-κB activation and preventing pathological inflammation [46]. Likewise, OTU family DUBs fine-tune antiviral signaling by trimming ubiquitin modifications on RIG-I and MAVS to calibrate interferon responses. Through these diverse mechanistic actions, DUBs operate as precisely tuned molecular rheostats that interpret and sculpt the ubiquitin code rather than indiscriminately block protein degradation [47], [48]. The principal mechanisms by which DUBs remove, trim, or edit ubiquitin chains are summarized in Fig. 3.

**Fig. 3:**
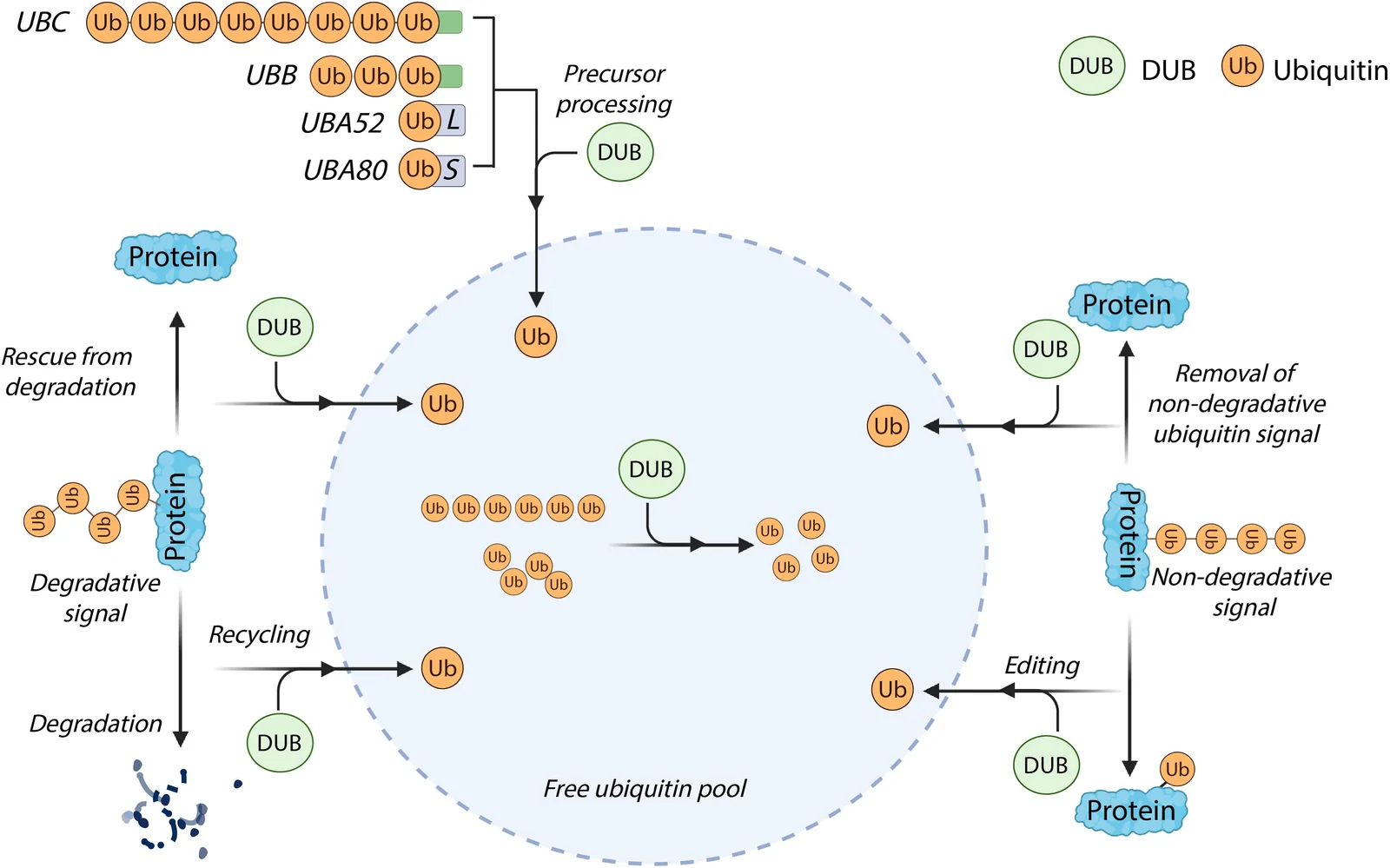
**Functional regulation of the ubiquitin system by DUBs:** Schematic representation of DUB-mediated ubiquitin removal and chain editing, including regulation of K48, K63, K11, and M1-linked ubiquitin signals, substrate fate, signaling, and ubiquitin recycling.

Given their central role in ubiquitin signaling, dysregulation of DUB activity contributes to numerous pathological conditions [49], particularly cancer, where aberrant stabilization of oncogenic proteins, impaired degradation of signaling mediators, and disruption of DNA repair collectively promote tumor initiation, progression, and therapeutic resistance [50], [51], [52]. More broadly, uncontrolled DUB activity drives pathological inflammation, metabolic reprogramming, replicative immortality, resistance to ferroptosis, and metastatic spread. Beyond oncology, DUB dysfunction contributes to neurodegenerative disorders (e.g., UCHL1 and Ataxin-3 in Parkinson's and spinocerebellar ataxia), autoimmune disease, and viral pathogenesis, reflecting their broad regulatory influence [49], [53], [54]. These findings have established DUBs as attractive therapeutic targets because selective modulation of individual DUBs offers greater pathway specificity than global proteasome inhibition. Consequently, defining the structural basis of substrate recognition and context-dependent DUB regulation remains essential for developing the next generation of precision therapeutics. Together, these mechanistic insights provide the foundation for understanding how dysregulated DUB activity drives tumor initiation, progression, and therapeutic resistance, which are discussed in the following sections.

Deubiquitinases (DUBs) have emerged as master regulators of cancer biology by controlling ubiquitin-dependent signaling pathways that govern protein stability, turnover, subcellular localization, and activity. Cancer cells frequently exploit DUB activity to stabilize oncogenic proteins or destabilize tumor suppressors, thereby sustaining proliferation, resisting apoptosis, and adapting to cellular stress [31], [55], [56]. Among the best-characterized examples is the p53-MDM2 signaling axis (Fig. 4), in which USP7, USP2, USP10, and other DUBs fine-tune p53 stability through differential regulation of MDM2 [18]. Overexpression of USP7 or USP2 stabilizes MDM2, promoting proteasomal degradation of p53 and suppression of apoptosis, whereas USP10 directly stabilizes wild-type p53 following DNA damage to facilitate cell-cycle arrest and genomic repair [18], [57], [58]. Although USP2 is frequently associated with oncogenic activity through MDM2 stabilization and suppression of p53, its biological effects depend on the substrate and signaling context. For example, USP2 also stabilizes VPRBP (DCAF1), a negative regulator of p53, and has been linked to regulation of the PD-L1 immune checkpoint [59]. In preclinical models, pharmacological inhibition of USP2 activated p53-dependent tumor suppression, and combining USP2 inhibition with PD-1 blockade produced substantial antitumor effects [60]. These findings illustrate the complexity of USP2-mediated signaling and suggest that its therapeutic targeting should account for tumor-specific molecular context and immune consequences. Likewise, USP9X promotes tumor cell survival by stabilizing the antiapoptotic protein MCL-1, contributing to resistance against chemotherapy and targeted therapies, particularly in hematological malignancies. Collectively, these findings demonstrate how DUB-mediated regulation of key oncogenic and tumor-suppressive pathways determines cancer cell fate and therapeutic responsiveness.

**Fig. 4:**
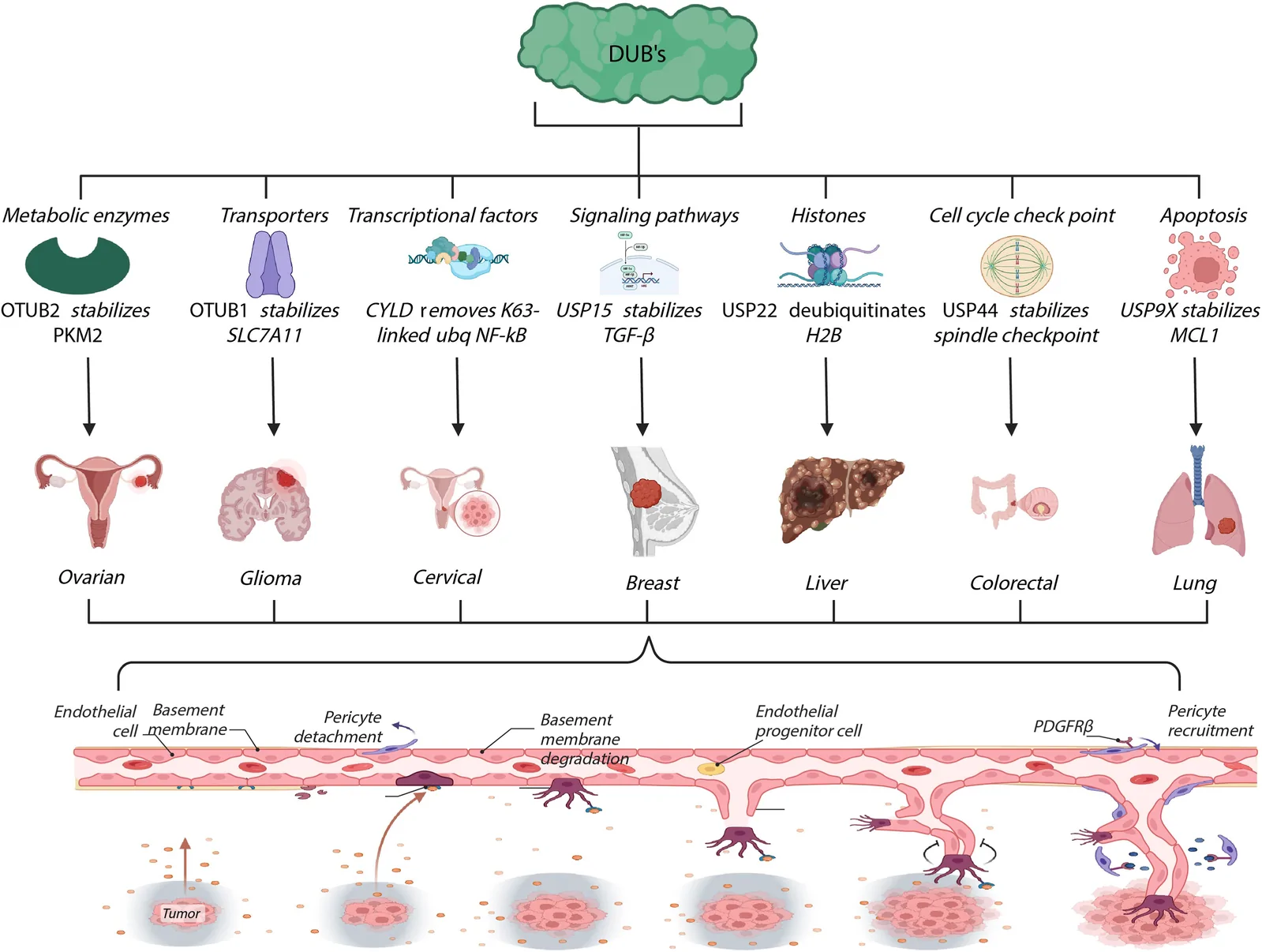
**Roles of DUBs in cancer progression and tumor regulation:** Schematic overview of representative oncogenic and tumor-suppressive DUBs and their involvement in cancer-associated processes, including cell survival, apoptosis, DNA damage repair, epigenetic regulation, immune signaling, epithelial–mesenchymal transition, metabolism, and metastasis.

Maintenance of genome integrity is essential for preventing malignant transformation, and numerous DUBs contribute to this process by regulating DNA repair pathways, replication stress responses, and chromatin dynamics. Among the best-characterized examples, USP1, USP47, and USP7 stabilize key DNA repair factors, including FANCD2, FANCI, CHK1, and DNA polymerase β, thereby promoting efficient repair of DNA damage and enabling cancer cells to survive genotoxic stress induced by chemotherapy or radiotherapy [61], [62], [63]. Beyond DNA repair, several DUBs regulate chromatin organization and transcriptional programs that influence tumor progression. As highlighted in Fig. 4, the tumor suppressor BAP1 deubiquitinates histone H2A to maintain chromatin homeostasis and regulate gene expression, and its loss is associated with aggressive malignancies including mesothelioma, uveal melanoma, renal cell carcinoma, and cholangiocarcinoma [64], [65], [66]. USP47 has also been implicated in cancer progression through the regulation of oncogenic signaling in colorectal cancer, USP47-mediated deubiquitination and stabilization of Yes-associated protein (YAP) has been associated with tumor progression, highlighting a potential link between ubiquitin-dependent protein stabilization and pro-tumorigenic transcriptional signaling. These findings broaden the relevance of USP47 beyond genome maintenance and support further investigation of its context-dependent functions in cancer [67]. In contrast, USP22, a catalytic component of the SAGA transcriptional coactivator complex, promotes transcriptional programs associated with proliferation, stemness, and tumor progression, and forms part of the well-described "death-from-cancer" gene signature. Together, these observations demonstrate that DUBs regulate cancer not only by controlling protein stability but also by reshaping the epigenetic landscape and transcriptional networks that govern tumor evolution.

Beyond regulating tumor growth and genome stability, DUBs play critical roles in metastatic dissemination by controlling epithelial–mesenchymal transition (EMT), cell migration, invasion, and interactions with the tumor microenvironment. DUBs such as OTUB1, USP11, USP7, USP10, and USP22 stabilize key EMT-associated transcription factors, including Snail, Slug, and β-catenin, thereby promoting epithelial plasticity, invasive behavior, and metastatic colonization of distant organs (Fig. 4) [68], [69]. JOSD2 has also been implicated in breast cancer metastasis. A recent study reported that JOSD2 expression was associated with metastatic disease and poor prognosis, and demonstrated that JOSD2 promotes breast cancer cell metastasis by removing polyubiquitin chains from SMAD4, thereby stabilizing this mediator of TGF-β signaling [70]. These findings identify JOSD2 as a potential contributor to metastatic progression and suggest that its effects on cancer depend on the substrates and signaling pathways engaged in a particular tumor context. STAMBPL1 (also known as AMSH-LP), a JAMM-family deubiquitinase, has been implicated in hepatocellular carcinoma (HCC) progression. A study reported that STAMBPL1 promotes HCC cell proliferation and metastatic behavior by deubiquitinating and stabilizing TNF receptor-associated factor 2 (TRAF2), thereby enhancing WNT, PI3K, and NF-κB signaling [71]. These findings identify STAMBPL1 as a potential regulator of oncogenic signaling and liver cancer progression and highlight the importance of distinguishing its functions from those of the related JAMM-family DUB STAMBP. DUBs also exert profound effects on antitumor immunity by regulating inflammatory signaling pathways and immune checkpoint stability. Negative regulators of NF-κB signaling, including CYLD, A20, OTULIN, and USP21, maintain immune homeostasis by editing K63- and M1-linked ubiquitin chains on signaling molecules such as TRAF6, RIPK1, and NEMO. Dysregulation of these DUBs promotes chronic inflammation and tumor progression. In addition, USP7 and USP22 stabilize the immune checkpoint protein PD-L1, thereby suppressing T-cell-mediated cytotoxicity and facilitating tumor immune evasion [52], [72]. Recent evidence further implicates JOSD2 in immune evasion in colorectal cancer. JOSD2 was shown to remove K27-linked ubiquitin chains from cyclic GMP-AMP synthase (cGAS), suppressing its enzymatic activity and limiting cGAS-STING pathway activation [73]. This mechanism promoted an immunosuppressive tumor microenvironment by favoring M2-like macrophage polarization. In preclinical models, pharmacological inhibition of JOSD2 restored cGAS-STING signaling and reduced colorectal tumor growth, highlighting a potential therapeutic link between DUB activity, innate immune sensing, and tumor immune escape. Collectively, these findings establish DUBs as central regulators of both metastatic progression and the dynamic interactions between tumor cells and the immune microenvironment, further highlighting their therapeutic relevance.

The diverse roles of DUBs in regulating oncogenic signaling, genome stability, epigenetic remodeling, metastasis, and tumor immune evasion underscore their importance as promising therapeutic targets in cancer. Unlike conventional proteasome inhibitors, which globally disrupt protein degradation and are frequently associated with systemic toxicity, selective targeting of individual DUBs offers the opportunity to modulate disease-relevant signaling pathways with greater precision. Consequently, considerable efforts have focused on developing DUB-directed therapeutic strategies, including small-molecule inhibitors targeting USP7, USP14, UCHL5, USP1, and other clinically relevant DUBs, together with emerging targeted protein degradation technologies such as PROTACs and related approaches [74], [75], [76].

Although significant progress has been achieved, challenges including substrate redundancy, context-dependent DUB functions, and the development of highly selective inhibitors continue to limit clinical translation. Nevertheless, rapid advances in structural biology, medicinal chemistry, and targeted protein modulation are accelerating the development of next-generation DUB-based therapeutics. The following section discusses current therapeutic strategies targeting DUBs, recent advances in inhibitor development, and the remaining challenges for translating DUB biology into precision oncology**.**

## 4 DUBs in cancer hallmarks

The diverse molecular functions of deubiquitinases (DUBs) ultimately converge on the canonical hallmarks of cancer (see Table: 2, Table: 3). Rather than acting as regulators of isolated signaling pathways, DUBs integrate multiple oncogenic processes by coordinating protein stability, signal transduction, transcriptional regulation, metabolic adaptation, and immune responses. Importantly, individual DUBs can influence several hallmarks simultaneously, reflecting the interconnected nature of ubiquitin-dependent signaling in cancer [31]. As summarized in Fig. 5 and Table 4, DUBs contribute to nearly every hallmark of cancer. They sustain proliferative signaling and overcome growth suppression by regulating key oncogenic and tumor suppressor pathways, including the p53-MDM2 axis, c-Myc, EGFR, AKT, and Cyclin D1 [57], [74], [75], [76], [77]. DUBs further promote tumor cell survival by stabilizing antiapoptotic proteins such as MCL-1 and BCL-xL while preserving proteostasis through proteasome-associated DUBs including USP14 and UCHL5 [52], [78], [79], [80]. DUBs also regulate metabolic reprogramming, angiogenesis, and replicative immortality through coordinated control of mitochondrial function, HIF-1α signaling, VEGF expression, telomere maintenance, and cancer stem cell homeostasis [81], [82], [83], [84], [85].

**Table: 2:** DUBs with oncogenic functions in cancer.

DUB	Mechanism	Effect	Cancer Type
USP7	Stabilizes MDM2 and suppresses p53 activity	Promotes cell survival and proliferation	Leukemia, Lymphoma, Prostate, Breast
USP9X	Stabilizes MCL-1 antiapoptotic protein	Enhances resistance to apoptosis	Hematologic malignancies, Lung, Pancreatic
USP22	Deubiquitinates H2B via SAGA complex	Drives stemness, metastasis, poor prognosis	Colorectal, Liver (HCC), Breast
USP28	Stabilizes MYC and NOTCH1 transcription factors	Supports tumor growth and transformation	Lung squamous carcinoma, Colorectal
USP1	Regulates FANCD2/ID1 DNA repair axis	Promotes DNA repair-mediated therapy resistance	Breast, Ovarian, Osteosarcoma
USP14	Regulates proteasome-associated substrate turnover	Enables proteotoxic stress tolerance	Multiple myeloma, Breast, Lung
UCHL1	Stabilizes metastasis-associated proteins (PAK1, β-catenin)	Facilitates invasion and metastasis	Breast, Ovarian, Melanoma
OTUB1	Inhibits E2 ligase–dependent ubiquitination controlling RhoA	Enhances migration, invasion, EMT	Prostate, Breast, Colorectal
USP15	Stabilizes TGF-β signaling components	Enhances EMT and metastatic dissemination	Glioblastoma, Breast, Ovarian
USP10	Stabilizes mutant p53 and AMPK pathway	Supports survival of mutant-p53 tumors	Pancreatic, Colon, Lung
USP44	Controls spindle checkpoint regulation	Overexpression leads to chromosomal instability	Colorectal, Breast
BRCC36 (BRCC3)	Part of BRCA1-A DNA repair complex	Promotes HR repair and therapy resistance	Breast, Ovarian (BRCA-mutant cancers)
PSMD14	Removes K63-linked ubiquitin chains from PKM2. This reduces the fraction of tetrameric PKM2, shifts equilibrium toward dimer/monomer forms.	Reduced pyruvate kinase (PK) enzymatic activity but enhanced nuclear translocation of PKM2	Ovarian cancer
OTUB2	Reported to deubiquitinate PKM2, antagonizing ubiquitination by certain E3 ligases (e.g. Parkin), thereby enhancing PKM2 activity	Promotes glycolysis and malignant growth in colorectal cancer, linking metabolic reprogramming to tumor progression.	colorectal cancer
JOSD2	Deubiquitinates and stabilizes SMAD4	Promotes metastatic progression	Breast cancer
STAMBPL1	Deubiquitinates and stabilizes TRAF2, activating WNT, PI3K, and NF-κB signaling	Promotes proliferation and metastatic behavior	Hepatocellular carcinoma
USP47	Deubiquitinates and stabilizes YAP	Supports oncogenic signaling and colorectal cancer progression	Colorectal cancer

**Table: 3:** DUBs with tumor-suppressive functions in cancer.

DUB	Mechanism	Effect	Cancer Type
CYLD	Removes K63-linked ubiquitin from NF-κB activators	Restrains NF-κB signaling, prevents inflammation-driven tumorigenesis	Skin, Liver, Multiple myeloma
A20 (TNFAIP3)	Deubiquitinates TRAF proteins, terminates TNF-α/NF-κB signaling	Limits chronic inflammation and malignant progression	Lymphoma, Leukemia
OTUD5	Stabilizes and protects p53	Promotes tumor suppressor activity and apoptosis	Breast, Colorectal
BAP1	H2A deubiquitination; chromatin remodeling	Supports transcriptional repression and DNA damage response	Mesothelioma, Uveal melanoma, Renal cell carcinoma
USP10	Deubiquitinates wild-type p53	Stabilizes p53, induces apoptosis under stress	Colon, Lung, Breast
USP29	Stabilizes p53 by deubiquitination	Promotes DNA damage response and tumor suppression	Leukemia, Lung
USP16	Deubiquitinates H2A to regulate chromatin structure	Supports cell-cycle checkpoints and limits proliferation	Leukemia, Breast
USP13	Deubiquitinates PTEN and other tumor suppressors	Enhances PTEN stability, inhibits PI3K/AKT signaling	Ovarian, Breast
OTUD1	Removes K48-linked ubiquitin from specific substrates	Prevents oncogenic protein accumulation	Breast, Gastric
USP3	Deubiquitinates H2A/H2B at DNA damage sites	Enhances genome stability, limits tumorigenesis	Breast, Colon
USP36	Deubiquitinates nucleolar proteins, maintains ribosome biogenesis	Tumor suppressor in specific contexts, prevents uncontrolled proliferation	Liver, Pancreatic

**Fig. 5:**
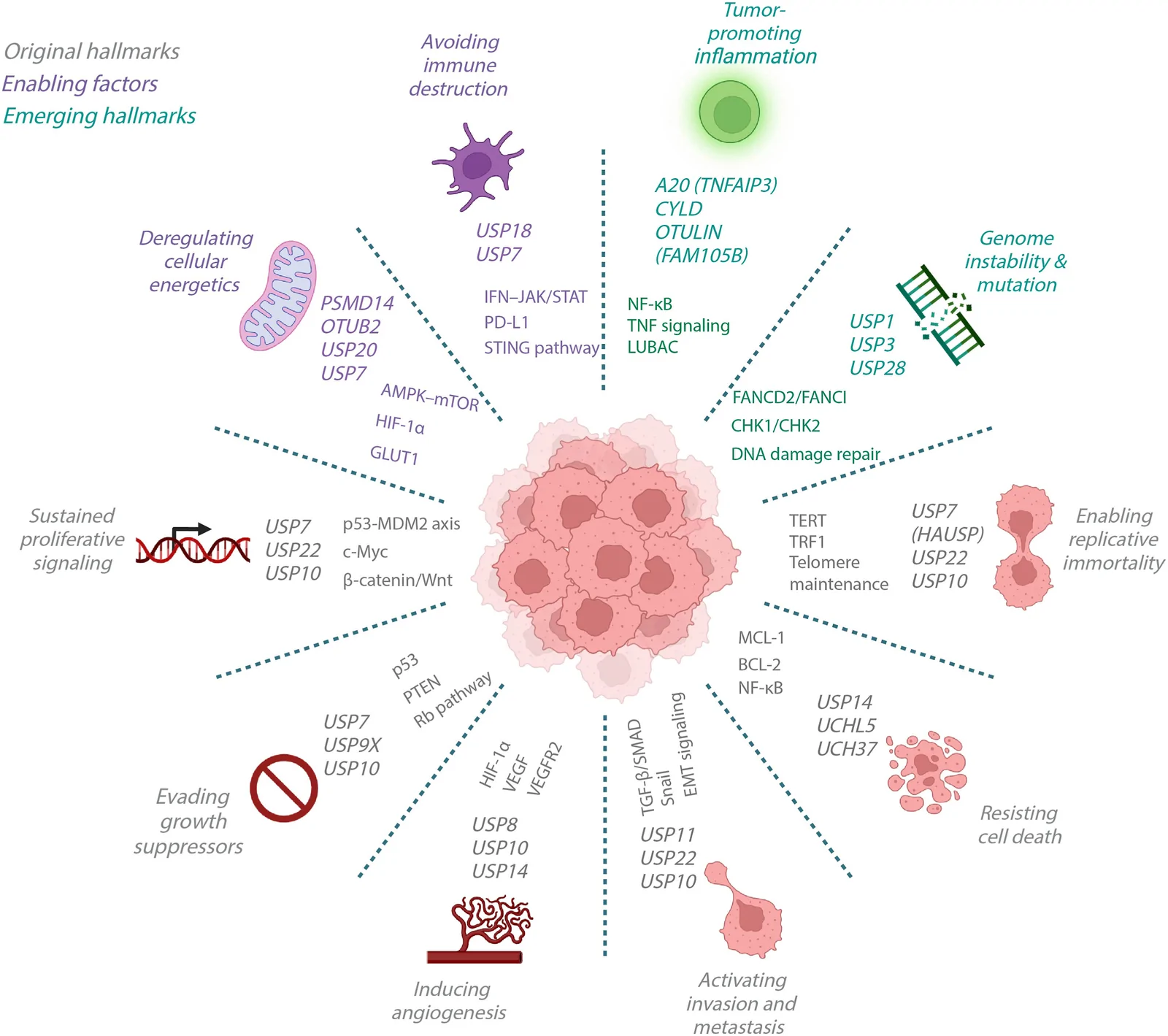
**Deubiquitinases regulate multiple hallmarks of cancer:** Schematic overview of the hallmarks of representative DUBs and their key substrates or signaling pathways associated with cancer hallmarks, including proliferation, survival, genome stability, metabolism, immune evasion, inflammation, angiogenesis, invasion, and metastasis.

**Table 4:** DUBs aligned with cancer hallmarks, substrates, and reported DUB-targeting compounds.

Cancer Hallmark	DUB	Key Substrate/Pathway Target	Functional Outcome in Cancer	Known Inhibitors	References
Sustaining proliferative signaling	USP7	MDM2/p53 axis; PTEN; FOXO4	Stabilizes MDM2, p53 degradation, cell proliferation	P5091, P22077, FT671, GNE-6640, GNE-6776	[86], [87], [88], [89]
	STAMBPL1	TRAF2; WNT, PI3K, and NF-κB signaling	Promotes HCC progression	No validated inhibitor reported	[71]
Evading apoptosis	USP9X	MCL-1	Stabilizes MCL-1, apoptosis resistance; chemoresistance	WP1130 (partial USP9X inhibitor) OR Degrasyn	[19], [90]
Resisting cell death & proteotoxic stress	USP14	Proteasome substrate trimming	Enhances proteasome activity, helps survival under stress	IU1, IU1-47, VLX1570	[91], [92], [93]
	UCHL5 (UCH37)	Proteasome-associated substrates	Controls proteasomal degradation & stress tolerance	b-AP15, VLX1570	[94], [95]
Genome stability & DNA repair	USP1	FANCD2, FANCI, PCNA	Supports Fanconi anemia pathway, DNA repair, chemo-resistance	ML323	[96], [97]
	USP3	Histone H2A/H2B; checkpoint proteins	Controls chromatin structure and DNA damage response	No inhibitor reported	
	Dual USP25/USP28 inhibitor	MYC, CHK2, 53BP1	Maintains replication stress tolerance and MYC stability	AZ1	[98], [99]
	USP47	YAP	Supports oncogenic signaling and colorectal cancer progression	P22077	[67]
EMT & metastasis	USP11	Snail	Stabilizes EMT transcription factors metastasis	Mitoxantrone	[100], [101], [102]
	USP22	SAGA complex, PD-L1	Supports EMT, invasion, and stemness	Emerging lead molecules (preclinical)	
	USP10	β-catenin, Smad4	Enhances EMT and migration	Spautin-1	[103], [104]
	JOSD2	SMAD4/TGF-β signaling	Promotes breast cancer metastasis	HY041004	[70], [105]
Immune evasion	USP18	ISG15 deconjugation; IFNAR–JAK–STAT signaling	Primarily an ISG15-specific isopeptidase and negative regulator of type I IFN signaling; suppresses antitumor interferon responses and may impair MHC-I-mediated immune surveillance	No selective inhibitor yet	[106], [107]
Evading Growth Suppressors	USP7	Deubiquitinates MDM2 and p53	Stabilizes MDM2, degradation of p53, loss of cell cycle arrest	P5091, P22077, FT671, FT827	[108], [109]
	USP9X	Stabilizes β-catenin & MCL-1	Enhances Wnt& survival signaling	WP1130	[110], [111]
	USP10	Stabilizes mutant p53; regulates PTEN	Supports proliferation, survival	Spautin-1	[112]
Inducing Angiogenesis	USP8	Stabilizes VEGFR2 by preventing ubiquitin-mediated degradation	Enhances VEGF signaling and endothelial cell proliferation	DUBs-IN-2, DUBs-IN-3	[113]
	USP10	Deubiquitinates and stabilizes HIF-1α	Promotes hypoxia-driven VEGF expression	Spautin-1	[114]
Deregulating Cellular Energetics	PSMD14	Deubiquitinates PKM2 (K63-linked)	Promotes PKM2 dimerization, Warburg effect & glycolysis	Capzimin	[115], [116]
	OTUB2	Stabilizes PKM2 via deubiquitination	Enhances glycolytic flux & tumor growth	No selective inhibitor reported	
	USP20	Binds and stabilizes PKM2	Enhances glycolysis & metabolic plasticity	Unclear/early discovery stage	
	USP7	Stabilizes PGC-1β & metabolic transcription factors	Increases oxidative phosphorylation & lipid metabolism	P5091, FT671, P22077	[115], [116]
Tumor-Promoting Inflammation	A20 (TNFAIP3)	Deubiquitinates K63 ubiquitin chains from RIP1, TRAF2, NEMO	Negative regulator of NF-κB; loss leads to chronic inflammation and protumor microenvironment	None selective available	
	CYLD	Cleaves K63/M1 ubiquitin chains on TRAF2, NEMO, RIPK1	Loss-of-function, uncontrolled NF-κB, chronic inflammation, enhanced proliferation	No small-molecule inhibitor known	
	OTULIN (FAM105B)	Removes M1-linked ubiquitin from LUBAC complex	Restrains NF-κB signaling; dysregulation contributes to inflammatory tumor microenvironment	None available	
Enabling Replicative Immortality	USP7 (HAUSP)	Stabilizes p53–MDM2 axis, affects telomere maintenance via TRF1 regulation	Supports cell survival & replicative capacity; allows bypass of senescence	P5091, P22077, FT671, FT827	[89], [117], [118]
	USP22	Part of SAGA complex; regulates c-Myc transcription & telomerase expression	Enhances telomerase (hTERT) activity and cancer stemness properties	None selective	
	USP10	Stabilizes SIRT6 and p53	Promotes telomere chromatin integrity & replicative longevity	Spautin-1	[119], [120]

DUBs also facilitate tumor progression by coordinating epithelial-mesenchymal transition, metastatic dissemination, genome maintenance, and immune escape. Representative DUBs stabilize EMT-associated transcription factors, regulate DNA repair pathways, and modulate inflammatory signaling networks, while others promote immune evasion through stabilization of immune checkpoint proteins such as PD-L1 [46], [63], [103], [121], [122], [123]. Tumor-promoting inflammation and immune evasion are regulated by distinct but interconnected DUB-dependent pathways. A20 (TNFAIP3), CYLD, and OTULIN act as important brakes on NF-κB signaling by editing K63- or M1-linked ubiquitin chains on signaling components such as RIPK1, NEMO, and the LUBAC complex, thereby limiting excessive inflammatory signaling [46], [124], [125], [126]. Loss or dysregulation of these DUBs can promote persistent NF-κB activation, chronic inflammation, and an immunosuppressive tumor microenvironment [127], [128]. In contrast, USP18 functions primarily as an ISG15-specific isopeptidase and a negative regulator of type I interferon signaling, with minimal conventional ubiquitin deubiquitinase activity in human cells [129], [130]. Its immunomodulatory effects therefore largely reflect its ability to attenuate IFNAR-JAK-STAT signaling, thereby influencing antitumor interferon responses; emerging evidence also links USP18 to impaired MHC-I-mediated immune surveillance in cancer [106], [131]. Together with DUBs such as USP7 and USP22, these pathways illustrate how DUB dysregulation can reshape inflammatory signaling, antigen presentation, and tumor immune interactions. Importantly, many DUBs including USP7, USP22, USP10, and OTUB1 participate in multiple hallmarks simultaneously, highlighting their function as interconnected signaling hubs rather than regulators of individual biological processes. This widespread functional convergence provides a compelling biological rationale for therapeutic targeting of DUBs in precision oncology.

Fig. 5 illustrates the extensive involvement of DUBs across the hallmarks of cancer, whereas Table 4 summarizes representative DUBs, their principal substrates, associated hallmarks, and currently available pharmacological inhibitors. Together, these observations emphasize that selective modulation of DUB activity has the potential to simultaneously disrupt multiple oncogenic programs, providing the foundation for the therapeutic strategies discussed in the following section.

## 5 Therapeutic targeting of DUBs

Given their central roles in oncogenic signaling, DNA repair, apoptosis, metabolic adaptation, and immune regulation, DUBs have emerged as attractive targets for precision cancer therapy. Unlike conventional proteasome inhibitors, which globally impair protein degradation and are associated with significant toxicity, selective DUB inhibitors offer the opportunity to modulate specific ubiquitin-dependent signaling pathways while preserving overall proteostasis [22], [132]. As illustrated in Fig. 6, multiple therapeutic strategies have been developed to target DUBs at transcriptional, post-translational, and catalytic levels.

**Fig. 6:**
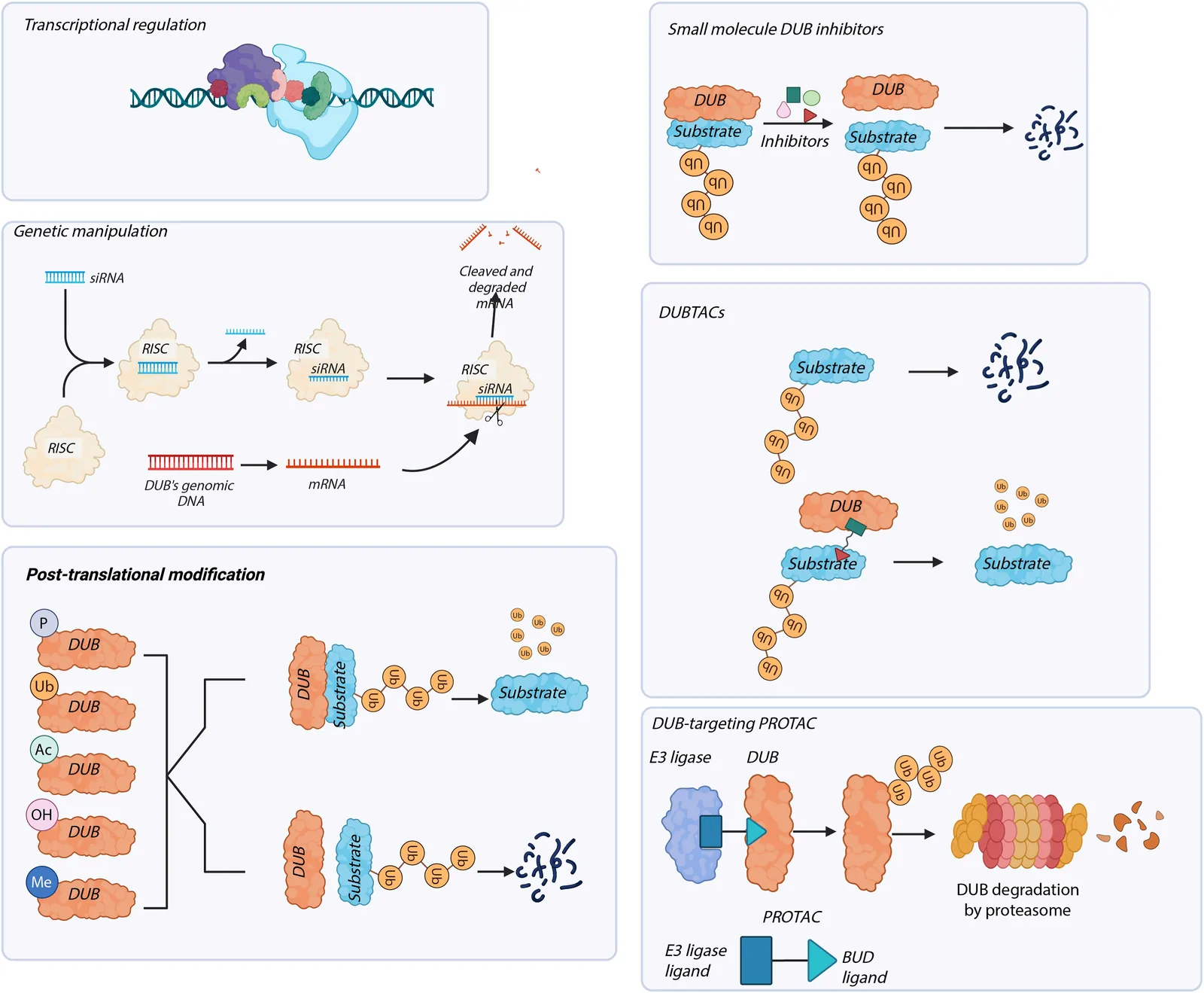
**Therapeutic strategies for targeting DUBs:** Schematic overview of approaches used to modulate DUBs, including transcriptional and genetic regulation, post-translational control, small-molecule inhibition, DUBTACs and DUB-targeting PROTACs.

Small-molecule inhibitors currently represent the most advanced therapeutic strategy for pharmacological targeting of DUBs (Table 5). Selective inhibition of oncogenic DUBs such as USP7, USP9X, USP14, USP22, and UCHL5 has demonstrated strong antitumor efficacy by destabilizing proteins essential for cancer cell survival and therapy resistance [86], [133], [134]. USP7 inhibitors including FT671, P5091, GNE-6776, and XL177A suppress MDM2-mediated degradation of p53, thereby restoring apoptotic signaling. USP9X inhibitors such as WP1130 and EOAI3402143 lead to MCL-1 destabilization, sensitizing lymphomas and solid tumors to chemotherapeutics. Inhibition of proteasome-associated DUBs (USP14 and UCHL5) using IU1, b-AP15, VLX1570, and related compounds induce proteotoxic stress and preferentially kill malignancies with high proteostasis demand, including myeloma and metastatic tumors. Additional inhibitors targeting USP1 (e.g., ML323, increasing sensitivity to DNA-damaging agents) and USP28/USP25 (e.g., AZ1, destabilizing MYC and CHK2) highlight the expanding scope of catalytic DUB blockade [86], [94], [135]. Importantly, the therapeutic success of DUB inhibition will likely depend not only on inhibitor potency but also on achieving sufficient target selectivity and identifying patient populations most likely to benefit from DUB-directed therapies (see Table 6).

**Table 5:** List of DUB inhibitors.

Inhibitor	Target DUB	Mechanism	Disease Model	Development Status
VLX1570	USP14, UCHL5	Dual inhibitor of proteasome-associated DUBs	Multiple myeloma, AML	Phase I clinical trial (terminated due to dose-limiting toxicity)
FT-7051	USP7	Selective USP7 inhibitor	Metastatic castration-resistant prostate cancer	Phase I clinical trial (terminated)
MTX325	USP30	Selective USP30 inhibitor	Parkinson's disease (PD)	Phase I clinical trial (Recruiting)
KSQ-4279 (RO7623066/RG6614)	USP1	Selective USP1 inhibitor	Advanced solid tumors	Phase I clinical trial (Completed) (NCT05240898)
WP1130	USP9X, USP5, USP14, UCHL5	Inhibits several DUBs, induces apoptosis	CML, lymphoma	Preclinical
P5091	USP7	Blocks p53 deubiquitination; stabilizes p53	Multiple myeloma, colorectal cancer	Preclinical
b-AP15	USP14, UCHL5	Inhibits proteasome-associated DUBs; triggers apoptosis	Solid tumors, lymphoma	Preclinical
PR-619	Pan-DUB inhibitor	Broad-spectrum DUB inhibition	Breast cancer, leukemia	Preclinical
FT671	USP7	Highly selective USP7 inhibitor; disrupts MDM2–p53 axis	Colorectal, melanoma	Preclinical
FT827	USP7	Irreversible USP7 inhibitor	AML, lymphoma	Preclinical
XL188	USP7	Selective small-molecule inhibitor	Prostate cancer, leukemia	Preclinical
HBX 41,108	USP7	Disrupts USP7 substrate interactions	Neuroblastoma, leukemia	Preclinical
Spautin-1	USP10, USP13	Promotes Beclin-1 degradation; blocks autophagy	Breast cancer, prostate cancer	Preclinical
IU1	USP14	Selective inhibition of USP14 at proteasome	Solid tumors (preclinical)	Preclinical
ML323	USP1	Inhibits USP1-UAF1 complex; affects DNA repair	Ovarian cancer, leukemia	Preclinical
AZ1	USP25, USP28	Disrupts oncogenic DUB signaling	Colorectal cancer	Preclinical
Vialinin A	USP5	Inhibits USP5-mediated protein stabilization	Melanoma	Preclinical

**Table 6:** Emerging targeted protein modulation strategies involving DUBs.

Name/Molecule	DUB (or Target)	Mechanism	Notes
MS6869 (and related USP7-DUBTACs)	USP7	DUB-recruiting chimera recruits USP7 to a protein-of-interest (POI) to deubiquitinate & stabilize the POI (e.g. mutant CFTR)	Representative USP7-based DUBTAC that stabilizes a target protein.
USP7-based DUBTACs (various linker versions)	USP7	DUBTACs built using non-covalent USP7 ligands, designed to stabilize POIs subjected to ubiquitination and degradation	Demonstrates feasibility of therapeutic protein stabilization via DUB recruitment.
USP7-PROTAC-1 (tool compound)	USP7 itself	Classical PROTAC: recruits an E3 ligase to ubiquitinate USP7 for degradation not exactly “DUB-recruiting,” but DUB is the degradation target	One of the first reported DUB degraders, showing that DUBs themselves can be degraded by PROTAC technology.

Beyond their direct effects on tumor cell survival, several DUBs have emerged as important regulators of antitumor immunity, highlighting new opportunities for cancer immunotherapy. Among these, USP18 has attracted considerable attention due to its ability to negatively regulate type I interferon (IFN) signaling by suppressing JAK-STAT pathway activation [136]. Elevated USP18 expression in multiple cancers contributes to immune evasion by attenuating interferon-mediated antitumor responses and reducing immune cell activation [131], [137]. Consequently, pharmacological inhibition or genetic depletion of USP18 has been shown to enhance interferon signaling, increase tumor immunogenicity, and improve the efficacy of immune checkpoint blockade in preclinical models [106], [138]. Other DUBs, including CYLD, A20 (TNFAIP3), and OTULIN, also regulate NF-κB signaling, inflammatory responses, and immune homeostasis, further emphasizing the diverse immunomodulatory roles of DUBs within the tumor microenvironment [46]. These findings suggest that selective targeting of immune-regulatory DUBs may not only suppress tumor growth directly but also potentiate antitumor immune responses, providing a strong rationale for combining DUB-targeted therapies with immunotherapeutic strategies.

Beyond conventional catalytic inhibition, recent advances in targeted protein modulation have expanded the therapeutic landscape of DUBs. Emerging technologies aim not only to inhibit DUB enzymatic activity but also to manipulate ubiquitin signaling with greater precision by selectively stabilizing or degrading disease-associated proteins. These innovative approaches offer new opportunities to overcome the limitations of traditional small-molecule inhibitors, including limited selectivity, off-target toxicity, and adaptive resistance mechanisms [139], [140], [141]. Among these strategies, deubiquitinase-targeting chimeras (DUBTACs) have emerged as a promising platform for therapeutic protein stabilization. Unlike proteolysis-targeting chimeras (PROTACs), which recruit E3 ubiquitin ligases to induce target protein degradation, DUBTACs recruit endogenous DUBs to remove ubiquitin chains from selected proteins, thereby preventing their proteasomal degradation. The first proof-of-concept DUBTACs utilized OTUB1 as the recruited DUB to restore the stability of disease-relevant proteins, demonstrating the feasibility of targeted protein rescue [142]. Although still in the early stages of development, this strategy has considerable potential for restoring the function of tumor suppressors and other beneficial proteins that are pathologically destabilized in cancer. Conversely, DUB-directed PROTACs represent an emerging strategy to eliminate oncogenic DUBs themselves through ubiquitin-mediated degradation. Rather than inhibiting catalytic activity alone, these degraders can achieve sustained suppression of DUB function by removing the entire protein, potentially overcoming limitations associated with reversible inhibition and non-catalytic scaffold functions. Although only a limited number of DUB-targeting degraders have been reported to date, continued advances in structural biology, ligand discovery, and rational degrader design are expected to accelerate their development [74], [143].

Despite encouraging preclinical advances several, challenges continue to limit the clinical translation of DUB-targeted therapies. Achieving selectivity among structurally conserved DUB families, particularly within the USP family, remains difficult and increases the risk of off-target effects. In addition, functional redundancy among DUBs, context-dependent substrate recognition, and incomplete understanding of DUB biology complicate patient stratification and therapeutic response prediction. Continued advances in structural biology, AI-assisted molecular design, and activity-based profiling technologies are expected to accelerate the development of safer and more selective DUB-targeting therapeutics.

## 6 Targeted protein modulation strategies: DUBTACs and DUB-targeting PROTACs

Beyond conventional small-molecule inhibitors, targeted protein modulation technologies have emerged as innovative strategies for manipulating DUB-dependent signaling. These approaches extend therapeutic intervention beyond catalytic inhibition by enabling either selective stabilization or degradation of disease-associated proteins through engineered bifunctional molecules. Among these, DUBTACs and DUB-targeting PROTACs represent complementary platforms that exploit the ubiquitin-proteasome system to restore or eliminate specific proteins with high precision [139], [141], [144], [145].

DUBTACs (Deubiquitinase-Targeting Chimeras) represent a novel class of heterobifunctional molecules designed to rescue proteins that undergo pathological ubiquitin-mediated degradation [144], [146]. In contrast to conventional PROTACs, which promote target protein degradation, DUBTACs recruit endogenous deubiquitinases to selectively remove ubiquitin chains from target proteins, thereby preventing their proteasomal degradation and restoring protein stability [139]. Unlike conventional PROTACs, which depend on E3 ubiquitin ligases to promote target protein degradation, DUBTACs catalytically recruit endogenous deubiquitinases to remove ubiquitin chains and stabilize proteins. This complementary mechanism makes DUBTACs particularly attractive for restoring the function of destabilized tumor suppressors and other proteins of therapeutic interest, although its effectiveness will depend on the availability, catalytic competence, substrate recognition, and cellular localization of the recruited DUB. While potentially overcoming limitations associated with tissue-specific E3 ligase expression and degradation-dependent resistance mechanisms [147]. The first proof-of-concept DUBTACs utilized OTUB1 as the recruited DUB to stabilize disease-associated proteins, demonstrating the feasibility of targeted protein rescue through engineered deubiquitination [139], [140]. More recently, the first DUBTAC targeting the cancer-associated p53 Y220C mutant demonstrated successful reactivation and stabilization of mutant p53 through OTUB1 recruitment, providing the first in vivo proof-of-concept for DUBTAC-mediated restoration of tumor suppressor function [148]. Furthermore, emerging TF-DUBTAC platforms have expanded this concept by enabling selective stabilization of transcription factors that have traditionally been considered difficult to target, highlighting the growing versatility and therapeutic potential of targeted deubiquitination technologies [142]. However, these advances remain largely preclinical, and the generalizability of DUBTAC-mediated stabilization across different substrates and cellular contexts remains to be established. Although DUBTACs provide a potentially powerful strategy for selective protein stabilization, their therapeutic development remains at an early stage and important mechanistic and translational questions remain unresolved. An important mechanistic concern has emerged regarding USP7-based DUBTACs that use inhibitor-derived USP7 ligands [140]. Since these ligands were originally designed to inhibit USP7, their use as recruiting ligands raises an apparent paradox, as catalytic inhibition could impair the deubiquitination required for target stabilization. Nevertheless, USP7-based DUBTACs have been shown to promote USP7-dependent substrate stabilization and reduce ubiquitination. Possible explanations include residual catalytic activity, ligand-dependent conformational or spatial effects, or productive ternary-complex formation, although these mechanisms remain unresolved. More broadly, DUBTACs face limitations including limited in vivo validation, incompletely defined substrate specificity, and potential off-target protein stabilization, warranting further mechanistic and in vivo investigation.

DUB-targeting PROTACs represent a complementary strategy that aims to selectively eliminate oncogenic deubiquitinases rather than inhibit their catalytic activity [74], [143]. Unlike DUBTACs, these bifunctional molecules recruit an E3 ubiquitin ligase to induce ubiquitination and subsequent proteasomal degradation of the target DUB. By removing the entire protein, DUB-targeting PROTACs have the potential to abolish both catalytic and non-catalytic scaffold functions, thereby providing more sustained suppression of oncogenic signaling than reversible enzyme inhibition alone. Although this approach remains in its early stages, proof-of-concept studies have demonstrated the feasibility of targeted degradation of cancer-associated DUBs, highlighting its promise as a next-generation therapeutic strategy [94], [141], [148], [149]. Continued advances in structural biology, ligand discovery, and rational degrader design are expected to accelerate the development of selective DUB-targeting PROTACs with improved efficacy and safety profiles [141], [144]. Collectively, DUBTACs and DUB-targeting PROTACs represent complementary approaches that expand the therapeutic landscape beyond conventional DUB inhibition by enabling selective stabilization or degradation of disease-associated proteins. Although still largely in the preclinical stage, these technologies hold considerable promise for advancing precision oncology through programmable manipulation of the ubiquitin-proteasome system.

## 7 Clinical translation & future challenges

Despite remarkable advances in understanding DUB biology and the development of selective inhibitors, successful clinical translation of DUB-targeted therapies remains in its infancy. Although inhibitors targeting USP7, USP14, USP9X, and other oncogenic DUBs have demonstrated encouraging preclinical efficacy in hematological malignancies and solid tumors, [150], [151], several biological and pharmacological challenges continue to limit their clinical application, one of the foremost challenges is achieving sufficient target selectivity. The high degree of structural conservation within the catalytic domains of many DUB families, particularly the USP family, complicates the development of inhibitors that selectively target individual enzymes while minimizing off-target toxicity [152]. Additionally, the limited structural resolution of DUB catalytic and regulatory domains restricts rational drug design and structure-guided optimization [153]. A further limitation is that many DUB-substrate relationships have been established using overexpression or ectopic-expression systems, which may generate non-physiological interactions or exaggerate substrate stabilization. Consequently, relatively few reported DUB-substrate pairs have been comprehensively validated at endogenous levels using complementary genetic, biochemical, and functional approaches. Establishing whether a proposed substrate represents a physiologically relevant dependency, rather than an interaction observed under experimental overexpression, remains an important challenge for interpreting DUB function and identifying actionable therapeutic vulnerabilities. Functional redundancy within the ubiquitin-proteasome system leads to compensatory signaling and context-dependent substrate regulation, diminishing therapeutic response, while drug resistance mechanisms are emerging as significant obstacles [154], analogous to those observed with kinase inhibitors. Another major barrier is the difficulty in identifying predictive biomarkers of response, which hinders patient stratification and clinical trial success. Furthermore, the context-dependent functions of DUBs which may act as either oncogenes or tumor suppressors, heighten the risk of unintended biological outcomes. The functional consequence of targeting a DUB may vary with tissue type, genetic background, subcellular localization, substrate availability, and the activity of compensatory pathways, making it difficult to extrapolate findings from one tumor model to another and complicating patient selection and therapeutic translation. Importantly, the clinical experience with DUB inhibitors has also exposed the gap between promising preclinical activity and therapeutic efficacy in patients. Several compounds that showed potent antitumor effects in experimental models have encountered limitations related to toxicity, pharmacokinetics, target selectivity, or insufficient therapeutic windows during clinical development. The clinical experience with proteasome-associated DUB inhibition, including VLX1570, which was discontinued because of dose-limiting toxicity, illustrates that strong mechanistic rationale and preclinical efficacy do not necessarily translate into a clinically tolerable treatment. These experiences underscore the need for rigorous target-engagement studies, pharmacodynamic biomarkers, and early evaluation of therapeutic windows during DUB inhibitor development. To address these limitations, next-generation strategies including allosteric modulators, covalent probes, and DUB-directed PROTACs/DUBTACs, coupled with advanced proteomics and structural biology, are expected to drive progress. Addressing these challenges will be essential for determining which DUB dependencies are physiologically relevant and for translating DUB modulation into safe, biomarker-guided clinical strategies.

## 8 Future opportunities

The rapid evolution of structural biology, chemical biology, and computational drug discovery is creating unprecedented opportunities to accelerate the clinical development of DUB-targeted therapeutics. High-resolution cryo-electron microscopy, X-ray crystallography, and quantitative proteomics are providing increasingly detailed insights into DUB catalytic mechanisms, substrate recognition, and regulatory interactions, thereby facilitating structure-guided inhibitor design and rational optimization. In parallel, artificial intelligence (AI)-assisted molecular modeling, virtual screening, and ligand design are expected to improve the discovery of highly selective DUB modulators with favorable pharmacological properties.

Therapeutic innovation is also expanding beyond conventional catalytic inhibition, Emerging protein-modulation platforms, including DUBTACs and DUB-targeting PROTACs, provide complementary approaches to conventional catalytic inhibition [144], [155]. These innovative approaches, together with biomarker-driven patient stratification and precision oncology frameworks, are expected to improve therapeutic selectivity while minimizing off-target toxicity. Furthermore, rational combination strategies integrating DUB-targeted agents with PARP inhibitors, immune checkpoint inhibitors, kinase inhibitors, or conventional proteasome inhibitors may enhance antitumor efficacy and overcome acquired resistance.

Another important opportunity lies in biomarker-guided and combination-based treatment. Integration of genomic dependency mapping, ubiquitin-signaling profiles, and multi-omics data could identify tumor-specific DUB dependencies and improve patient selection. Rational combinations with DNA-damage-response inhibitors, immune checkpoint inhibitors, kinase inhibitors, or proteasome-directed therapies may further exploit context-specific vulnerabilities and help overcome resistance.

## 9 Concluding remarks

Deubiquitinases have emerged as central regulators of protein homeostasis, signaling, genome stability, epigenetic regulation, metabolism, and tumor-immune interactions. Their ability to modulate both degradative and non-degradative ubiquitin signals enables DUBs to influence multiple cancer-associated processes and provides a strong rationale for therapeutic intervention.

Although substantial progress has been made in identifying cancer-associated DUB dependencies and developing pharmacological and targeted protein-modulation strategies, translation into effective therapies remains constrained by target selectivity, context-dependent DUB function, therapeutic toxicity, resistance, and limited predictive biomarkers. These challenges highlight the importance of validating DUB dependencies at physiological levels and integrating mechanistic studies with structural biology, chemical proteomics, and systems-level approaches.

Future progress will depend on defining which DUB dependencies are truly actionable in specific tumor contexts and developing biomarker-guided strategies to exploit them safely. Continued integration of molecular, structural, proteomic, and clinical approaches should help establish DUB-directed therapies as a more precise component of cancer treatment.

## CRediT authorship contribution statement

**Mohd Altaf Najar:** Writing – review & editing, Writing – original draft, Visualization, Supervision, Software, Resources, Project administration, Methodology, Investigation, Formal analysis, Data curation, Conceptualization. **Prashant Kumar Modi:** Writing – review & editing, Supervision. **Raja Rajeshwari:** Software.

## Ethics approval and consent to participate

Not applicable.

## Funding

No funding.

## Declaration of competing interest

The authors declare that they have no known competing financial or non-financial interests that could have appeared to influence the work reported in this paper.

## Data Availability

No data was used for the research described in the article.
